# Supplementary figures and images for: Identification of Key Differentially Expressed Genes in Arabidopsis thaliana Under Short- and Long-Term High Light Stress
Source: Int J Mol Sci. 2025 Aug 12;26(16):7790. doi: 10.3390/ijms26167790 (PMC12386182; doi:10.3390/ijms26167790)

## Dissimilarity

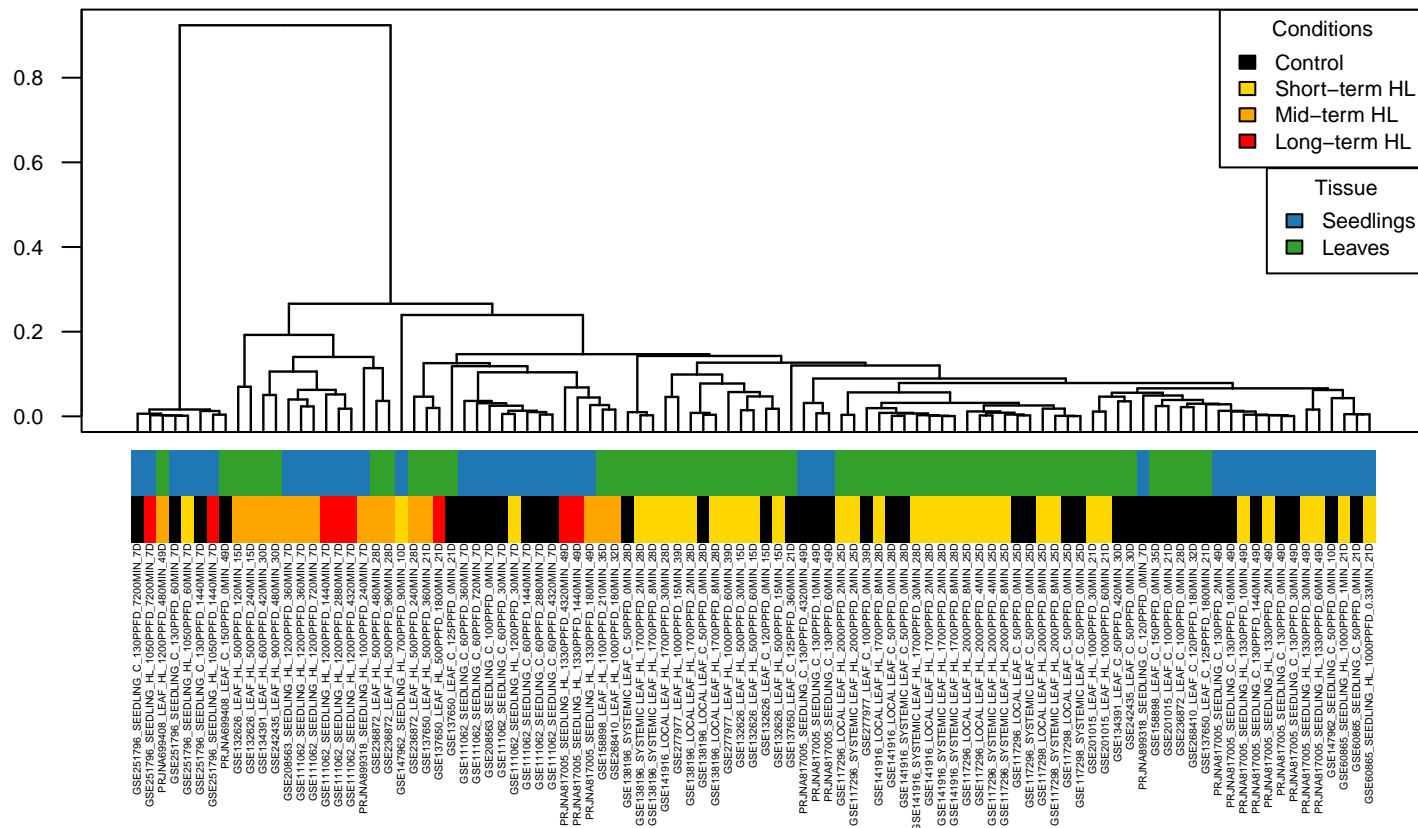

Supplement: Supplementary file 1 [file ijms-26-07790-s001.zip › Supplementary File S2.pdf]

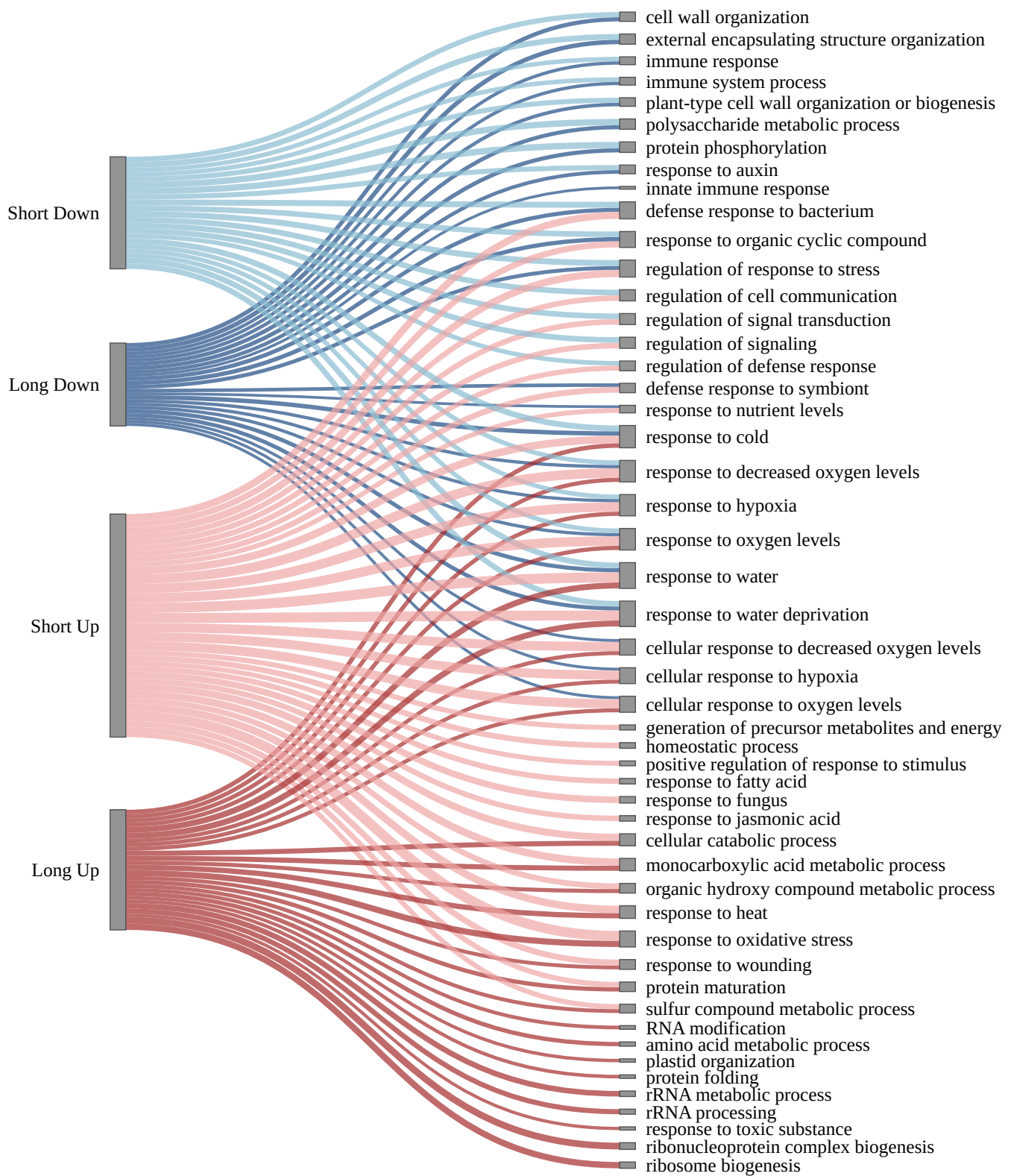

Supplement: Supplementary file 1 [file ijms-26-07790-s001.zip › Supplementary File S3.pdf]
