# Supplementary material for: Identification of Key Differentially Expressed Genes in Arabidopsis thaliana Under Short- and Long-Term High Light Stress
Source: Int J Mol Sci. 2025 Aug 12;26(16):7790. doi: 10.3390/ijms26167790 (PMC12386182; doi:10.3390/ijms26167790)
Supplement: Supplementary file 1 [file ijms-26-07790-s001.zip › Supplementary File S4.zip!/Antioxidant_heatmap_seedlings.pdf]

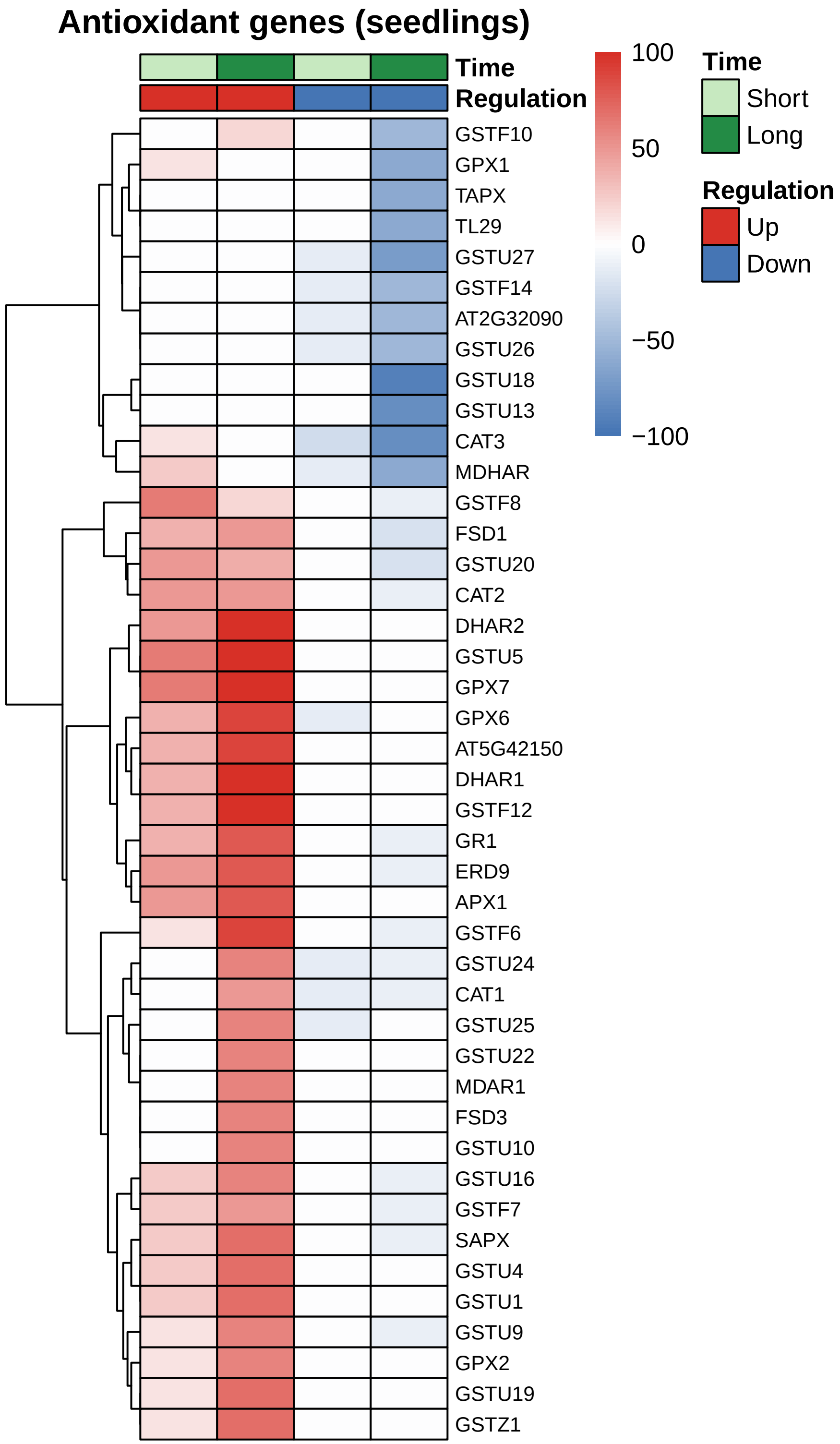
Antioxidant genes (seedlings)
Time
Regulation
100
50
0
−50
−100
Time
Short
Long
Regulation
Up
Down
GSTF10
GPX1
TAPX
TL29
GSTU27
GSTF14
AT2G32090
GSTU26
GSTU18
GSTU13
CAT3
MDHAR
GSTF8
FSD1
GSTU20
CAT2
DHAR2
GSTU5
GPX7
GPX6
AT5G42150
DHAR1
GSTF12
GR1
ERD9
APX1
GSTF6
GSTU24
CAT1
GSTU25
GSTU22
MDAR1
FSD3
GSTU10
GSTU16
GSTF7
SAPX
GSTU4
GSTU1
GSTU9
GPX2
GSTU19
GSTZ1
