# Supplementary material for: Identification of Key Differentially Expressed Genes in Arabidopsis thaliana Under Short- and Long-Term High Light Stress
Source: Int J Mol Sci. 2025 Aug 12;26(16):7790. doi: 10.3390/ijms26167790 (PMC12386182; doi:10.3390/ijms26167790)
Supplement: Supplementary file 1 [file ijms-26-07790-s001.zip › Supplementary File S4.zip!/BBX_heatmap_seedlings.pdf]

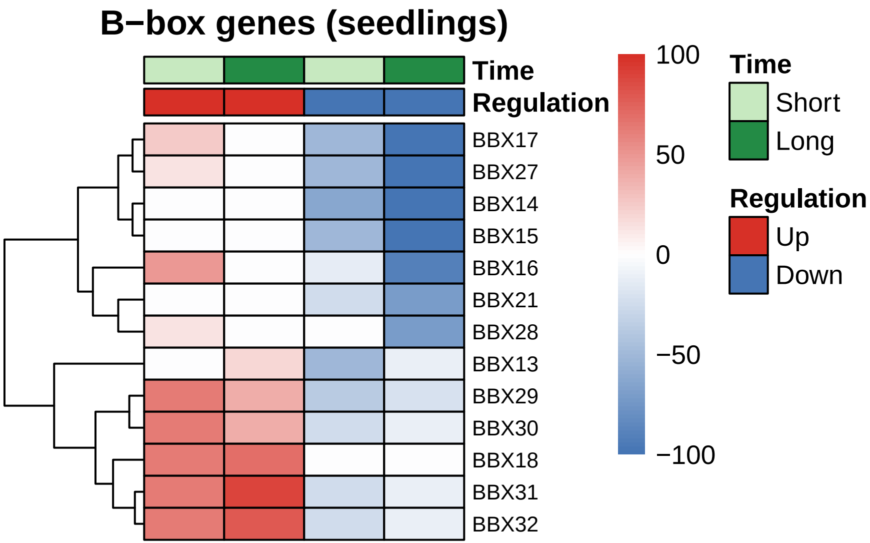

B−box genes (seedlings)
Time
Regulation
BBX17
BBX27
BBX14
BBX15
BBX16
BBX21
BBX28
BBX13
BBX29
BBX30
BBX18
BBX31
BBX32
100
50
0
−50
−100
Time
Short
Long
Regulation
Up
Down
