# Supplementary material for: Identification of Key Differentially Expressed Genes in Arabidopsis thaliana Under Short- and Long-Term High Light Stress
Source: Int J Mol Sci. 2025 Aug 12;26(16):7790. doi: 10.3390/ijms26167790 (PMC12386182; doi:10.3390/ijms26167790)
Supplement: Supplementary file 1 [file ijms-26-07790-s001.zip › Supplementary File S4.zip!/Chloroplast_organization_heatmap_seedlings.pdf]

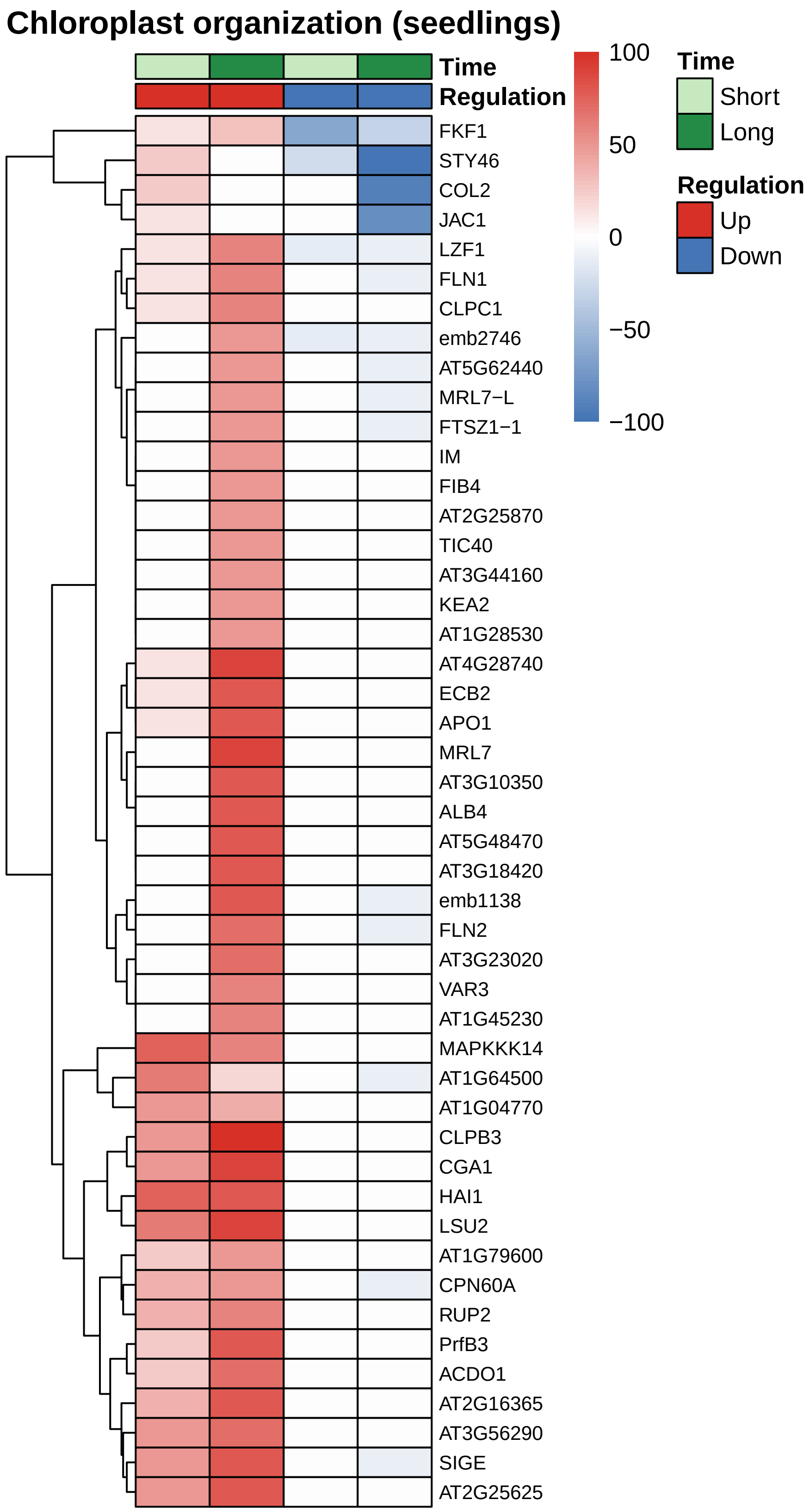

Chloroplast organization (seedlings)
Time
Regulation
FKF1
STY46
COL2
JAC1
LZF1
FLN1
CLPC1
emb2746
AT5G62440
MRL7−L
FTSZ1−1
IM
FIB4
AT2G25870
TIC40
AT3G44160
KEA2
AT1G28530
AT4G28740
ECB2
APO1
MRL7
AT3G10350
ALB4
AT5G48470
AT3G18420
emb1138
FLN2
AT3G23020
VAR3
AT1G45230
MAPKKK14
AT1G64500
AT1G04770
CLPB3
CGA1
HAI1
LSU2
AT1G79600
CPN60A
RUP2
PrfB3
ACDO1
AT2G16365
AT3G56290
SIGE
AT2G25625
100
50
0
−50
−100
Time
Short
Long
Regulation
Up
Down
