# Supplementary material for: Identification of Key Differentially Expressed Genes in Arabidopsis thaliana Under Short- and Long-Term High Light Stress
Source: Int J Mol Sci. 2025 Aug 12;26(16):7790. doi: 10.3390/ijms26167790 (PMC12386182; doi:10.3390/ijms26167790)
Supplement: Supplementary file 1 [file ijms-26-07790-s001.zip › Supplementary File S4.zip!/PhANGs_heatmap_seedlings.pdf]

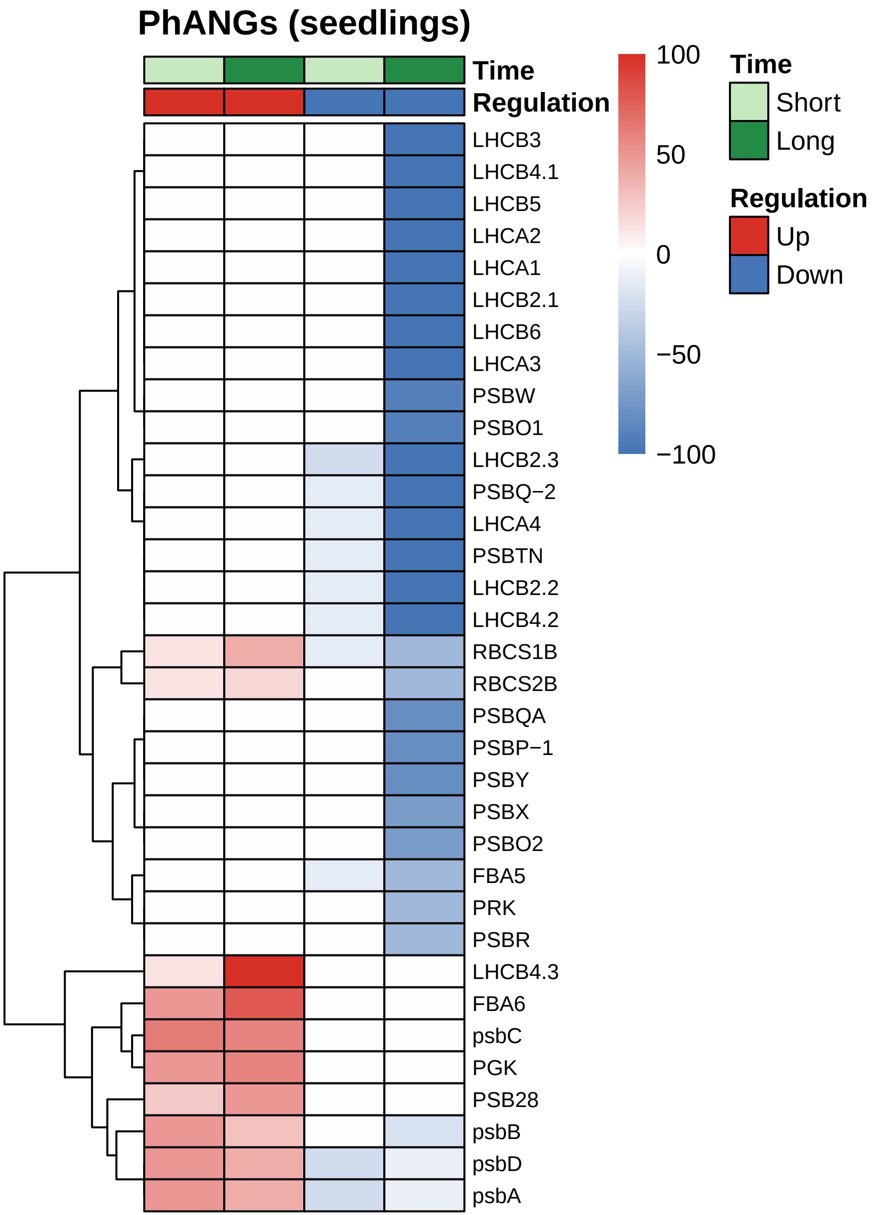

PhANGs (seedlings)
Time
Regulation
LHCB3
LHCB4.1
LHCB5
LHCA2
LHCA1
LHCB2.1
LHCB6
LHCA3
PSBW
PSBO1
LHCB2.3
PSBQ−2
LHCA4
PSBTN
LHCB2.2
LHCB4.2
RBCS1B
RBCS2B
PSBQA
PSBP−1
PSBY
PSBX
PSBO2
FBA5
PRK
PSBR
LHCB4.3
FBA6
psbC
PGK
PSB28
psbB
psbD
psbA
100
50
0
−50
−100
Time
Short
Long
Regulation
Up
Down
