# Supplementary material for: Identification of Key Differentially Expressed Genes in Arabidopsis thaliana Under Short- and Long-Term High Light Stress
Source: Int J Mol Sci. 2025 Aug 12;26(16):7790. doi: 10.3390/ijms26167790 (PMC12386182; doi:10.3390/ijms26167790)
Supplement: Supplementary file 1 [file ijms-26-07790-s001.zip › Supplementary File S4.zip!/Photomorphogenesis_heatmap_seedlings.pdf]

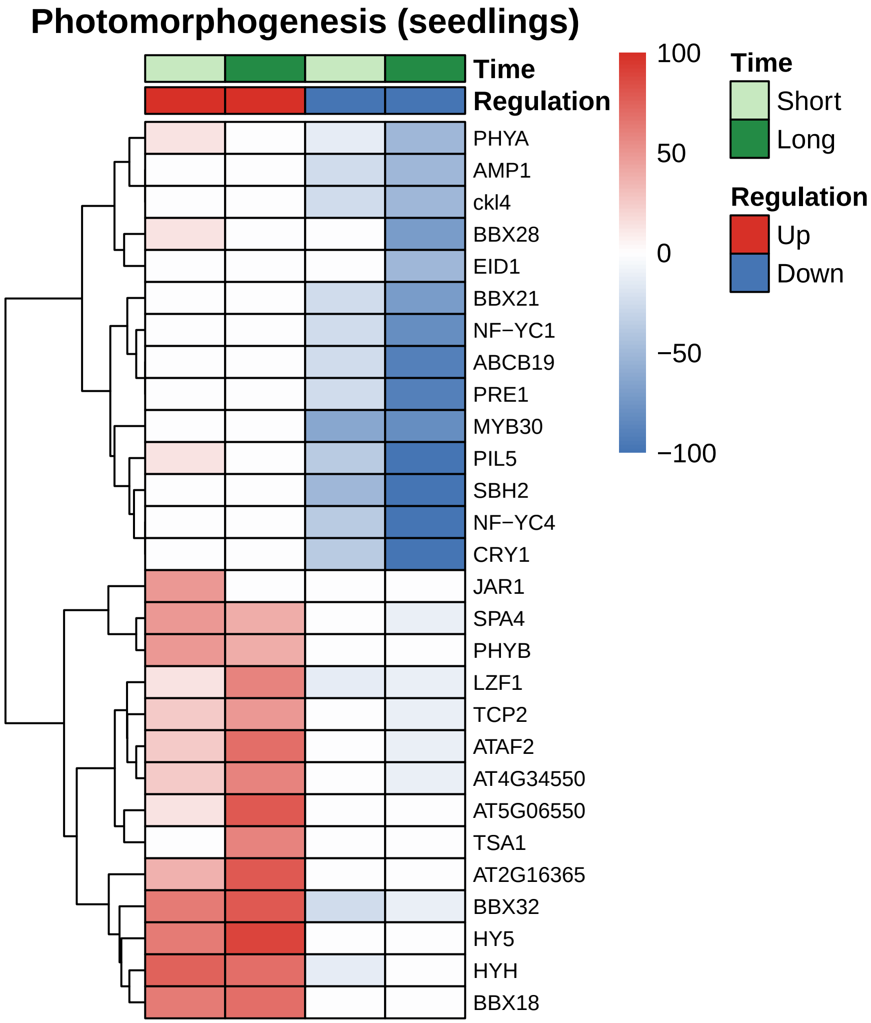

Photomorphogenesis (seedlings)
Time
Regulation
PHYA
AMP1
ckl4
BBX28
EID1
BBX21
NF−YC1
ABCB19
PRE1
MYB30
PIL5
SBH2
NF−YC4
CRY1
JAR1
SPA4
PHYB
LZF1
TCP2
ATAF2
AT4G34550
AT5G06550
TSA1
AT2G16365
BBX32
HY5
HYH
BBX18
100
50
0
−50
−100
Time
Short
Long
Regulation
Up
Down
