# Supplementary material for: Identification of Key Differentially Expressed Genes in Arabidopsis thaliana Under Short- and Long-Term High Light Stress
Source: Int J Mol Sci. 2025 Aug 12;26(16):7790. doi: 10.3390/ijms26167790 (PMC12386182; doi:10.3390/ijms26167790)
Supplement: Supplementary file 1 [file ijms-26-07790-s001.zip › Supplementary File S4.zip!/Photoreceptors_heatmap_seedlings.pdf]

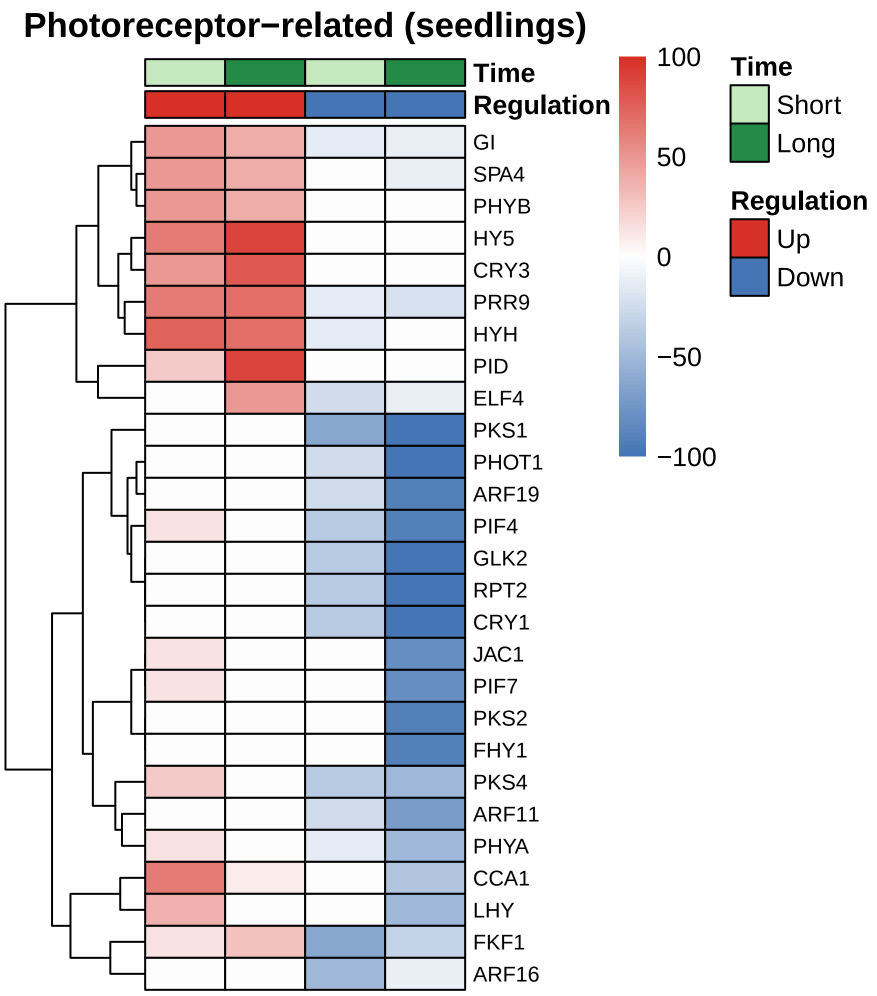
Photoreceptor−related (seedlings)
Time
Regulation
GI
SPA4
PHYB
HY5
CRY3
PRR9
HYH
PID
ELF4
PKS1
PHOT1
ARF19
PIF4
GLK2
RPT2
CRY1
JAC1
PIF7
PKS2
FHY1
PKS4
ARF11
PHYA
CCA1
LHY
FKF1
ARF16
100
50
0
−50
−100
Time
Short
Long
Regulation
Up
Down
