# Supplementary material for: Identification of Key Differentially Expressed Genes in Arabidopsis thaliana Under Short- and Long-Term High Light Stress
Source: Int J Mol Sci. 2025 Aug 12;26(16):7790. doi: 10.3390/ijms26167790 (PMC12386182; doi:10.3390/ijms26167790)
Supplement: Supplementary file 1 [file ijms-26-07790-s001.zip › Supplementary File S4.zip!/Redoxins_heatmap_seedlings.pdf]

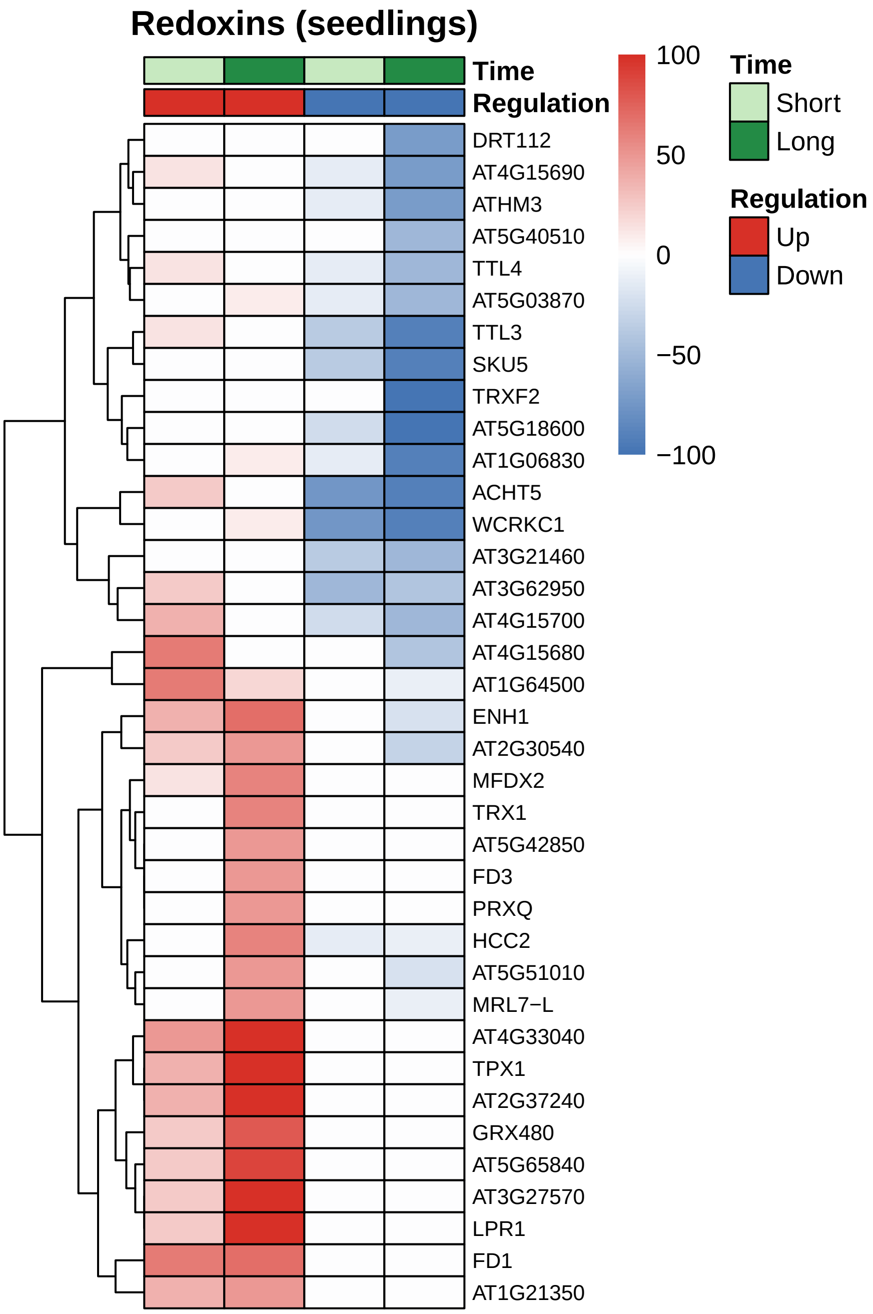

Redoxins (seedlings)
Time
Regulation
100
50
0
−50
−100
Time
Short
Long
Regulation
Up
Down
DRT112
AT4G15690
ATHM3
AT5G40510
TTL4
AT5G03870
TTL3
SKU5
TRXF2
AT5G18600
AT1G06830
ACHT5
WCRKC1
AT3G21460
AT3G62950
AT4G15700
AT4G15680
AT1G64500
ENH1
AT2G30540
MFDX2
TRX1
AT5G42850
FD3
PRXQ
HCC2
AT5G51010
MRL7−L
AT4G33040
TPX1
AT2G37240
GRX480
AT5G65840
AT3G27570
LPR1
FD1
AT1G21350
