# Supplementary material for: Identification of Key Differentially Expressed Genes in Arabidopsis thaliana Under Short- and Long-Term High Light Stress
Source: Int J Mol Sci. 2025 Aug 12;26(16):7790. doi: 10.3390/ijms26167790 (PMC12386182; doi:10.3390/ijms26167790)
Supplement: Supplementary file 1 [file ijms-26-07790-s001.zip › Supplementary File S4.zip!/TFs_heatmap_seedlings.pdf]

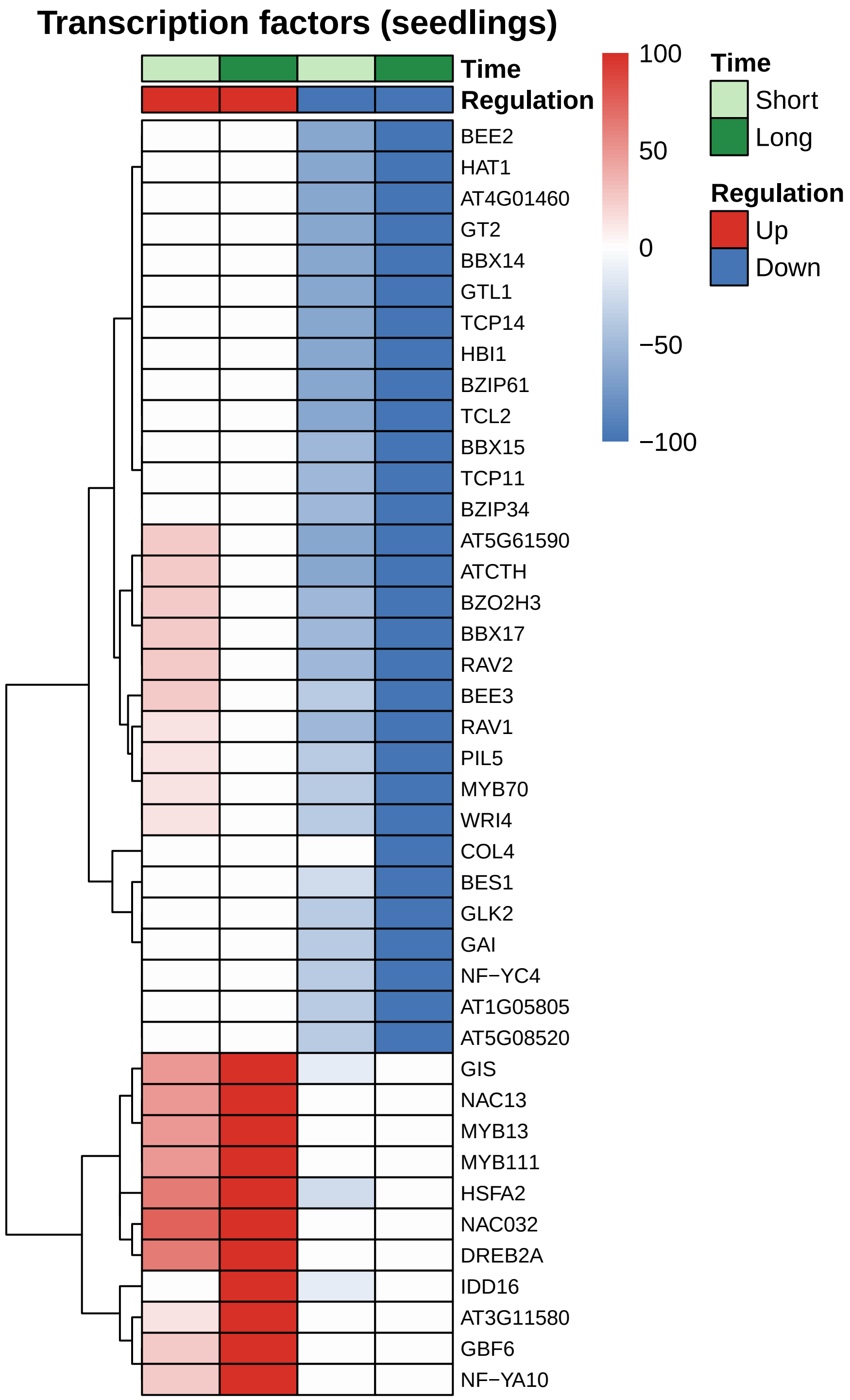
Transcription factors (seedlings)
Time
Regulation
BEE2
HAT1
AT4G01460
GT2
BBX14
GTL1
TCP14
HBI1
BZIP61
TCL2
BBX15
TCP11
BZIP34
AT5G61590
ATCTH
BZO2H3
BBX17
RAV2
BEE3
RAV1
PIL5
MYB70
WRI4
COL4
BES1
GLK2
GAI
NF−YC4
AT1G05805
AT5G08520
GIS
NAC13
MYB13
MYB111
HSFA2
NAC032
DREB2A
IDD16
AT3G11580
GBF6
NF−YA10
100
50
0
−50
−100
Time
Short
Long
Regulation
Up
Down
