# Supplementary material for: Identification of Key Differentially Expressed Genes in Arabidopsis thaliana Under Short- and Long-Term High Light Stress
Source: Int J Mol Sci. 2025 Aug 12;26(16):7790. doi: 10.3390/ijms26167790 (PMC12386182; doi:10.3390/ijms26167790)
Supplement: Supplementary file 1 [file ijms-26-07790-s001.zip › Supplementary File S5.zip!/GRN_long_seedlings.pdf]

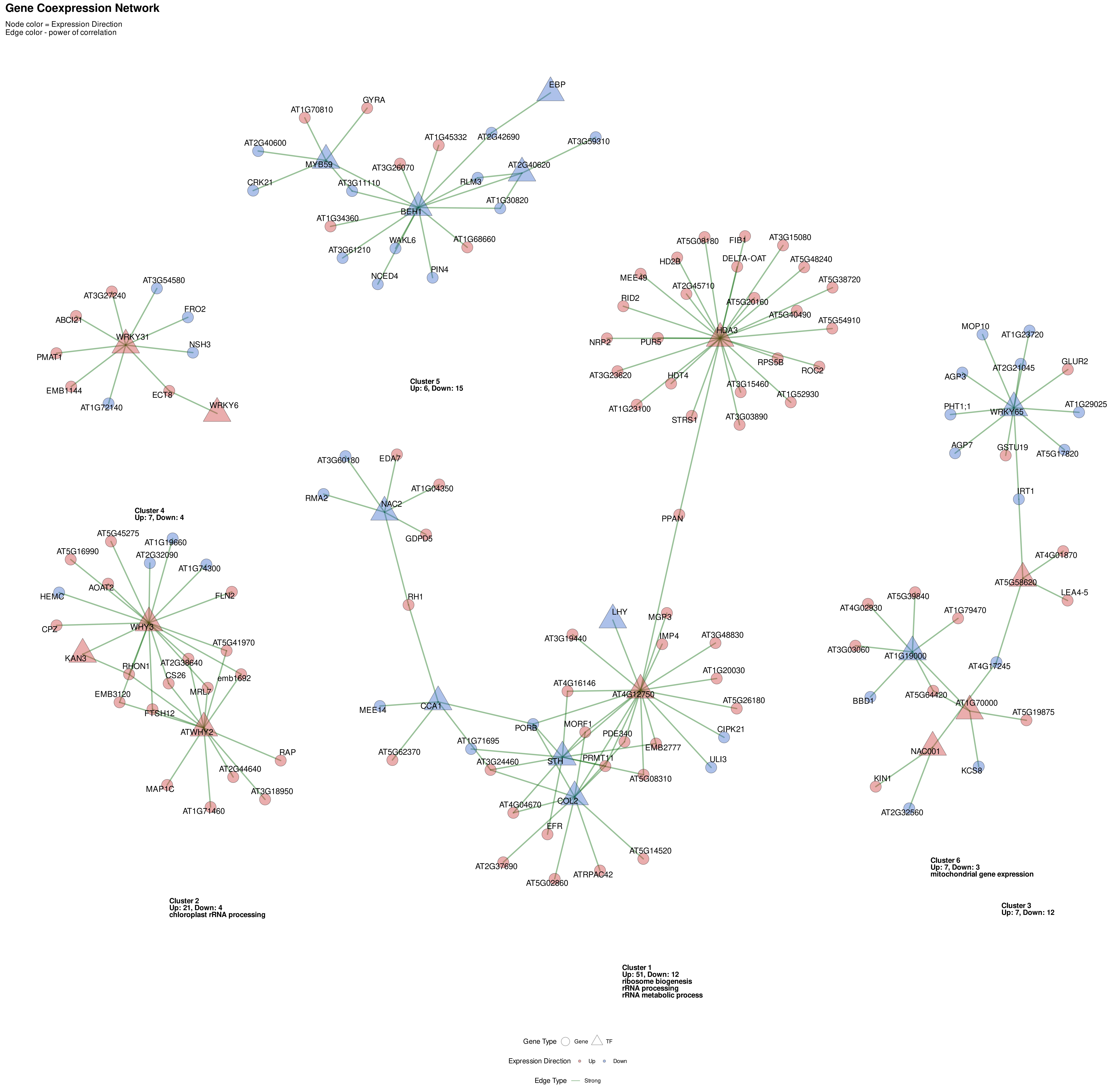

Gene Coexpression Network
Node color = Expression Direction
Edge color - power of correlation
EBP
GYRA
AT1G70810
AT1G45332
AT2G42690
AT3G59310
AT2G40600
MYB59
AT3G26070
AT2G40620
CRK21
AT3G11110
RLM3
AT1G30820
BEH1
AT1G34360
AT1G68660
WAKL6
AT3G61210
NCED4
PIN4
AT3G54580
AT3G27240
FRO2
ABCI21
WRKY31
NSH3
PMAT1
EMB1144
ECT8
AT1G72140
WRKY6
Cluster 5
Up: 6, Down: 15
AT5G08180
FIB1
AT3G15080
HD2B
DELTA-OAT
AT5G48240
MEE49
AT2G45710
AT5G38720
RID2
AT5G20160
AT5G40490
AT5G54910
NRP2
PUR5
HDA3
RPS5B
ROC2
AT3G23620
HDT4
AT3G15460
AT1G52930
AT1G23100
STRS1
AT3G03890
MOP10
AT1G23720
GLUR2
AGP3
AT2G21045
PHT1;1
WRKY65
AT1G29025
AGP7
GSTU19
AT5G17820
IRT1
AT3G60180
EDA7
AT1G04350
RMA2
NAC2
GDPD5
PPAN
Cluster 4
Up: 7, Down: 4
AT5G45275
AT1G19660
AT5G16990
AT2G32090
AT1G74300
AOAT2
HEMC
FLN2
CPZ
WHY3
KAN3
AT5G41970
RHON1
AT2G38640
CS26
emb1692
MRL7
EMB3120
FTSH12
ATWHY2
RAP
AT2G44640
MAP1C
AT3G18950
AT1G71460
RH1
LHY
MGP3
AT3G19440
IMP4
AT3G48830
AT1G20030
AT4G16146
AT4G12750
AT5G26180
MEE14
CCA1
PORB
MORF1
PDE340
EMB2777
CIPK21
AT5G62370
AT1G71695
STH
PRMT11
ULI3
AT3G24460
AT5G08310
COL2
AT4G04670
EFR
AT5G14520
AT2G37690
ATRPAC42
AT5G02860
AT4G01870
AT5G58620
LEA4-5
AT5G39840
AT4G02930
AT1G79470
AT3G03060
AT1G19000
AT4G17245
BBD1
AT5G64420
AT1G70000
AT5G19875
NAC001
KCS8
KIN1
AT2G32560
Cluster 2
Up: 21, Down: 4
chloroplast rRNA processing
Cluster 6
Up: 7, Down: 3
mitochondrial gene expression
Cluster 3
Up: 7, Down: 12
Cluster 1
Up: 51, Down: 12
ribosome biogenesis
rRNA processing
rRNA metabolic process
Gene Type
Gene
TF
Expression Direction
Up
Down
Edge Type
Strong
