# Supplementary material for: Identification of Key Differentially Expressed Genes in Arabidopsis thaliana Under Short- and Long-Term High Light Stress
Source: Int J Mol Sci. 2025 Aug 12;26(16):7790. doi: 10.3390/ijms26167790 (PMC12386182; doi:10.3390/ijms26167790)

Gene Coexpression Network

Node color = Expression Direction
Edge color - power of correlation

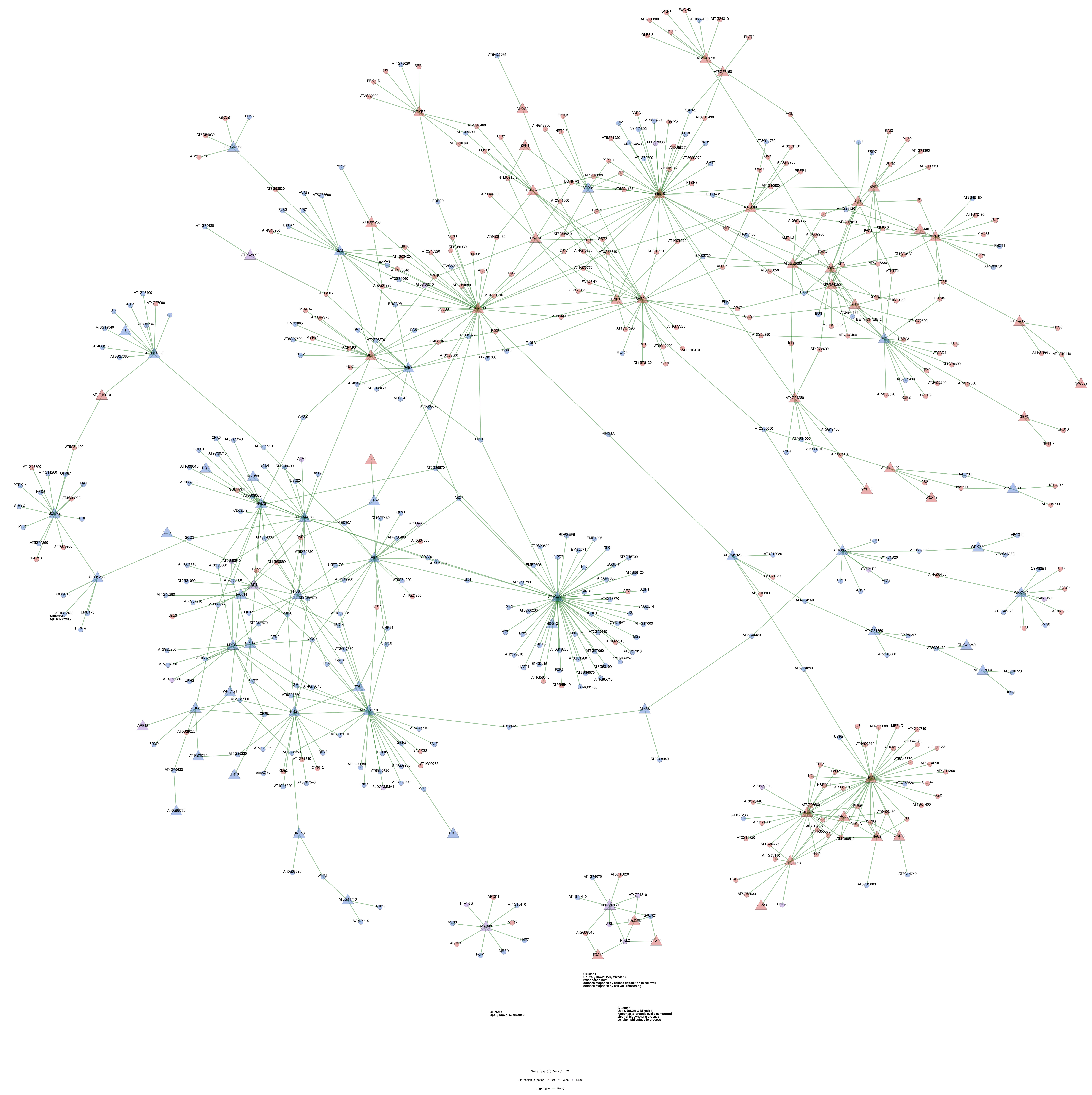

Supplement: Supplementary file 1 [file ijms-26-07790-s001.zip › Supplementary File S5.zip!/GRN_short_seedlings.pdf]
